# Method for the Culture of Mouse Pulmonary Microvascular Endothelial Cells

**Published:** 2017

**Authors:** Anita Kovacs-Kàsa, Matthew N Varn, Alexander D Verin, Joyce N Gonzales

**Affiliations:** 1 Vascular Biology Center, Augusta University Health, Augusta, USA; 2 Division of Pulmonary and Critical Care Medicine, Department of Internal Medicine, Augusta University Health, Augusta, USA

**Keywords:** Cell culture, Lung, Method, Microvascular endothelial cells, Murine pulmonary endothelial cells

## Abstract

Pulmonary microvascular endothelial cells (ECs) are integral to the alveoli-capillary barrier of the lung. The EC barrier integrity is known to be disrupted in severe lung diseases such as acute respiratory distress syndrome (ARDS), pneumonia and pulmonary edema. Mice are commonly used to model these diseases, dictating an increasingly high demand for murine ECs isolation and culture. Despite the significant number of protocols for the culture of various types of murine cells, the isolation of microvascular endothelial cells remains a challenging procedure. In our manuscript we developed adetailed step-by-step refined method for isolation murine pulmonary microvascular ECs for *in vitro* studies. We separated cells using platelet endothelial cell adhesion molecule antibody and characterized ECs with antibodies against intercellular adhesion molecule-1, acetylated-low density lipoprotein, and vascular endothelial (VE)-cadherin. Further, we confirmed microvascular origin of these cells using *Griffonia simplicifolia and Helix pomatia* (negative control) staining. Barrier properties of EC monolayer were characterized by conducting electric cell-substrate impedance sensing experiments with the edemagenic agents, lipopolysaccharide and nocodazole, and known barrier-protective agents, adenosine and sphingosine-1-phosphate. The described complete protocol provided consistent and reproducible results.

## Introduction

Pulmonary endothelial cells ECs are integral to the alveoli-capillary barrier of the lung. EC barrier is known to be disrupted in severe lung diseases such as acute respiratory distress syndrome ARDS, pneumonia and pulmonary edema. Mice are often employed to study these diseases. Cloned murine cells have many potential uses and applications in immunologic and physiologic studies [1]. Lung diseases are often studied with human pulmonary ECs *in vitro*. However, it is important to establish a reproducible method for isolating murine lung microvascular endothelial cells to examine the role and characteristics of the lung microvascular endothelium without the confounding environment of a variety cells found with *in vivo* murine lungs [2–4].

Commercially comparable cells are limited, and culture-based propagation of mouse pulmonary microvascular endothelial cells MPMVECs has proved difficult. Studies using live animals have elicited variability in results even in controlled conditions and in animals that are genetically identical [3]. One of the reasons for this variability in results is animal stress. One advantage of *in vitro* investigations is that cells can be studied in a controlled environment without the undue influences or stress that can occur in live animals. Animal models for lung disease are described in the Official American Thoracic Society Workshop Report: Features and Measurements of Experimental Acute Lung Injury in Animals [3]. However, there is a gap between human *in vitro* studies to murine *in vivo* studies. Despite human-mouse genetic homology of 95%, human *in vitro* studies have not been shown to be homologous to mouse *in vivo* studies. Theoretically, correlates in human studies can be developed. To bridge this gap we have utilized a method to culture MPMVECs.

Protocols for cultures of murine ECs are available, but obtaining good and consistent results remains a challenge. We reviewed and tested several protocols. The drawbacks in our experiments were limited growth of cells, early senescence, and low purity of cell type. Our protocol resulted in cells that could be used for multiple experiments: immunocytochemistry; quantitative reverse-transcription-polymerase chain reaction qPCR; electric cell-substrate impedance sensing ECIS; a complementary cDNA and RNA studies with qPCR for the toll like receptor 4 TLR4. We characterized cells as of endothelial origin using immunostaining with vascular endothelial cadherin VE-cadherin, acetylated-low density lipoprotein Ac-LDL, and intercellular adhesion molecule ICAM. As a negative control we stained NIH3T3 fibroblasts with VE cadherin. Microvascular features were characterized by *Griffonia simplicifolia* GS1 and *Helix pomatia* HPA negative control staining. Cell localization was identified by nuclear DAPI staining, if necessary. Functional responses of EC barrier were characterized using trans-endothelial electrical resistance TER measurements in ECIS assay with the well-known edemagenic agent, lipopolysaccharide LPS, and the microtubule inhibitor, nocodazole. Both agonists disrupt EC barrier *in vitro* and *in vivo* [5,6]. In addition, we characterize EC barrier strengthening using known EC barrier-protective agents, adenosine and sphingosine-1-phosphate [7,8].

Cell adhesion molecules are a family of closely related cell-surface glycoproteins. They are members of the immunoglobulin supergene family and expressed on ECs. Platelet endothelial cell adhesion molecule PECAM comprises a large portion of endothelial cell [9] intercellular junctions. In our method, PECAM is conjugated onto Dynabeads^®^ and used for separation of ECs. ICAM-1 is another fundamental member of the cell adhesion molecule family, and is also expressed on vascular ECs. ICAM-1 can be expressed on other cells, especially if stimulated by inflammatory cytokines, however is present in basal doses on ECs [10]. ECs can be separated with PECAM and characterized with ICAM-1, acetylated-low density lipoprotein, and VE-cadherin. EC microvascular origin can be further characterized with *Griffonia simplicifolia* and *Helix pomatia* negative control immunostaining [11].

Here, we describe a step-by-step method for the culture of MPMVECs. We have used this protocol for more than 2 years, and have obtained MPMVECs in requisite quantities for our experiments.

## Materials and Methods

### Ethical approval of the study protocol

The study protocol was approved by the Animal Care and Use Committee of Augusta University Augusta, GA, USA. The care and treatment of animals was according to guidelines set by the National Institutes of Health Bethesda, MD, USA.

### Animals

Mice age, 2–6 weeks were housed in cages with their mother before 3 weeks of age and independently after 3 weeks of age until the time of experimentation. They had free access to food and water in a temperature- and light-controlled room with a 12-h dark-light cycle. C57BL/6 mice were purchased from Charles River Laboratories Wilmington, MA, USA.

### Chemicals and reagents

The chemicals used for our method were purchased as shown in Table 1. The antibodies were those against VE-cadherin 160840; Cayman, Ann Arbor, MI, USA, ICAM-1 3422R-100; BioVision, Milpitas, CA, USA and Ac-LDL Sigma-Aldrich, St Louis, MO, USA. Secondary antibodies were Alexa Fluor^®^ 488 dye and Alexa Fluor^®^ 594dye Thermo Fisher Scientific, West Columbia, SC, USA. Lectin *Bandeiraea simplicifolia Griffonia simplicifolia* BS1 was obtained from Santa Cruz, CA, USA and lectin Helix pomatia Alexa Fluor 488conjugateLife Technologies, Carlsbad CA, USA and Texas Red Phalloidin was purchased from Thermo Fisher Scientific, Grand Island, NY, USA. Eight-well arrays were from Applied Biophysics Albany, NY, USA.

### Protocol description

All the material described below is considered sterile unless noted and accomplished under a sterile laminar flow hood. All solutions are passed through a 22-micron filter, placed in sterile containers and opened only under the laminar flow hood.

### Preparation of dynabeads

The Dynabead^®^ solution DBS is prepared using PBS with 0.1% BSA. Dynabeads^®^ Sheep Anti Rat IgG Invitrogen, Grand Island, NY, USA and are mixed by pipetting the beads up-and-down several times or gently shaking the container in which they are housed. Next the Dynabeads^®^ Sheep Anti Rat IgG 12 μL per mouse are suspended in 1 mL DBS in a 1.5 mL Eppendorf Tube-one tube for each mouse. The tube is then placed on a magnetic particle concentrator MPC Fisher Scientific, Pittsburgh, PA, USA and left for 60 seconds and while the tube remains on the magnet the supernatant is carefully removed and discarded. The tube is then removed from the MPC and another 1 mL DBS added this volume facilitates conjugation of Dynabeads^®^ and PECAM-1 during overnight rotation. The PECAM-1 antibody 10% of the Dynabead^®^ volume is added into the DBS and the Dynabead^®^/PECAM-1 solution incubated at 4 °C overnight on a slow rotator. The next day, the initial steps of the wash are repeated using 1 mL DBS, then wash 4 times and re-suspend the beads in the original amount with DBS and store at 4 °C until used.

### Preparation for dissection and isolation

Prior to the dissection, the incubatoris set to 37 °C and a large centrifuge to 4 °C. 0.2% collagenase type I with Hank’s balanced salt solution 1 × HBSS is prepared immediately prior to the mouse dissection. NOTE: We use 249 units/mg however each production lot of collagenase type I purchased is in various units per mg for that batch thus the units must be adjusted to the protocol. HBSS is pre-warmed to 37 °C and immediately before dissection, the collagenase added. The mixture is vortex-mixed and sterilized and when dissolved use a 0.22 μm syringe filter and 7 mL of solution and put into individual 15 mL tubes. One 15 mL tube is used for each set of mouse lungs. Additional HBSS × 1 is chilled at 4 °C and placed in a 60 mm dish to be used to clean and mince the lungs.

To prepare the tissue culture plates for the mouse lung cells we coat the plates to be used with 10 μg/cm^2^ fibronectin in H_2_O. The plates are kept under the tissue culture hood for 1 h, and then excess fibronectin content removed and the dish allowed to dry for a minimum of 30 minutes.

### Mouse dissection

Collagenase is prepared at this time as described above. Work quickly as collagenase cannot sit for > 30–45 min before beginning the digestion. Collagenase is dissolved in a warm water-bath < 5 min and left at room temperature under a laminar flow hood until required. In our experiments the mice are exposed to isoflurane in an inhalation chamber until no movement or righting reflex. Then are removed and quickly euthanized by cervical dislocation. The bodies are placed in a 10 cm petri dish on ice and sprayed with 70% alcohol, and transported to a sterile environment. Under the laminar flow hood, the arms and legs are pinned to a dissection table with 20-G needles.

Starting above the stomach, a section of tissue is lifted with the forceps and a skin incision made to gain access to the subdermal layer. Cutting upwards toward the neck using the forceps, the tissue is kept taut and the skin removed over the chest. We cut parallel to the rib cage to ensure the diaphragm is exposed. We then puncture the diaphragm using scissors. Next, we hold the xiphoid process with the forceps and cut vertically up the sternum, thereby splitting the ribs in half. Both sides of the rib cage are removed, getting as close to the lungs as possible without damaging them. The mouse is rotated 180°. Using another pair of forceps or the previous ones cleansed in 70% alcohol, the heart is lifted and cut along the spine, removing any connections on the dorsal side. The heart and lungs are then placed in the cold HBSS in the 60 mm dish. An “ideal” lung removal results in a pink pair of lungs. This procedure is repeated for other mice. We culture 4–8 lungs per experiment.

### Tissue dissection

Once all lungs are collected, debris and non-lung tissue trachea, aorta, heart are removed rapidly. Once all of the lungs are cleaned move one pair at a time into a clean 60 mm dish with 500 μL of cold HBSS. With the dish tilted toward forward so that the HBSS covers the lung use small sharp surgical scissors and mince the tissue into approximately 125 pieces. At that point transfer the minced tissue to one of the prepared 15 mL tubes containing 3 mL warm collagenase. Using a 1 mL pipette tip and the 4 remaining milliliters of collagenase, any residual lung tissue in the dish is transferred and placed in the 15 mL tube. The 15 mL tube now contains 7 mL of collagenase and one set of minced lungs. Repeat these steps rapidly for the other lungs.

The 15 mL tubes containing lungs may sit at room temperature until processing. When all lungs are prepared place them in an oven at 37 °C for 45 min with gentle rotation for tissue dissociation and ensure that the tube tops are tightened. Tubes are agitated every 10 min to prevent clumping. After 45 min, the dissociated tissue is passed through a 70 μM filter screen. The screen is washed with equal parts of isolation medium. The tubes are centrifuged at 600 × g for 8 min at 37 °C. The supernatant is discarded, the pellet re-suspended in 1 mL of base medium DMEM high glucose with 1× Pen/Strep, and transferred to a 1.5 mL Eppendorf tube. There is a 1.5 mL tube for each mouse.

### Cell isolation

PECAM-conjugated Dynabeads^®^ 12 μL are pipetted into each 1.5-mL tube and rotated for 1 h at 4 °C. After 1 h of rotation, the tubes are placed on the MPC for 1–2 min. Wash the beads five times with 1 mL of isolation medium. This is a vital step that needs attention. Washing too easily results in large amounts of contaminating cells and washing too harshly removes all ECs. The best option is to remove the supernatant, remove the tube from the MPC, re-suspend the beads with 1 mL of isolation medium, aspirate 1 mL and then, with the tip ¼″ from the top, expel the 1 mL towards the tube bottom with a moderate amount of force. This helps to separate any clumping and removes unwanted cells. During the final wash, the isolation medium is replaced with Endothelial Growth Media-2 EBM-2.

### Seeding density

We seed the cells using a 12-well plate and 1–2 mice per well. After the cells are at least 80% confluent they may be passaged up to a 60 mm or 10 cm dish for propagation. In our experience at the initial seeding if the larger cell culture plates are used the cells do not grow well. The growth medium is EBM-2 containing streptomycin 100 UI/mL and penicillin 0.01%. We use this medium in all murine cell cultures. The medium is changed the next day and thereby every other day. Cells are passaged using cell dissociation solution for 4–8 min. The action of cell dissociation solution is stopped by adding the growth medium or isolation medium, centrifuging the cells as described above and reconstituting in EBM-2 we do not use trypsin.

### Isolation of total RNA, reverse transcription polymerase chain reaction PCR, and real-time PCR

Total RNA was isolated from MPMVECs using Trizol reagent Pierce, Grand Island, NY, USA according to manufacturer instructions. The concentration and purity of RNA was determined by measuring the absorbance at 260 nm and 280 nm Ultrospec 3000; Pharmacia Biotech, Centerville, VA, USA. cDNA ≈ 4 μg was synthesized by reverse transcription with M-MLV reverse transcriptase Invitrogen, Carlsbad, CA, USA using oligo dT primers BioRad, Hercules, CA, USA. Real-time PCR was undertaken on a Step One Real-Time PCR System Applied Biosystems, Grand Island, NY, USA using iTaq Universal SYBR Green Supermix BioRad, 100 ng of cDNA and 1 μM of forward and reverse primers.

### Preparation of total cell lysates

Cells were grown to 90% confluence on 100 mm dishes and rinsed twice with ice-cold PBS. Lysis buffer 500 μL was added, supplemented with a 1× Halt Protease and Phosphatase Inhibitor Cocktail, and harvested on ice by scraping. The cell suspension was sonicated on ice twice for 10 s at 20% and centrifuged at 33368 χ g for 15 min at 4 °C. The supernatant was saved and protein concentration determined using a Bicinchoninic Acid Protein Assay kit Pierce. The homogenate was stored at −80 °C.

### Immunocytochemistry and immunofluorescence

MPMVECs were plated onto glass coverslips and grown to confluence. Cells were washed once with 1 × PBS containing Ca^2+^ and Mg^2+^ and fixed in 3.7% paraformaldehyde in 1 × PBS for 10 min at room temperature. Between each step, cells were rinsed thrice with 1 × PBS. Cells were permeabilized with 0.25% Triton X-100 in Tris-buffered saline with Tween 25 mM Tris·HCl, pH 7.5, 150 mM NaCl, 0.1% Tween 20 for 5 min at room temperature, and then blocked with 2% BSA in Tris-buffered saline with Tween for 1 hour at room temperature. Cells were incubated with the primary antibody ICAM-1 1:500 dilution rat anti-mouse monoclonal antibody 3422R-100; BioVision or VE-cadherin polyclonal antibody 1:500 dilution; Cayman Chemicals for 1 h and then with the secondary antibodies Alexa Fluor 488 goat anti-rabbit or Alexa Fluor 594 goat anti-mouse diluted in blocking solution for 1 h at room temperature.

#### *Griffonia* staining

After fixation, permabilization and blocking steps see above MPMVEC cultures were stained with specific fluorescein isothiocyanate FITC labeled lectin from *Bandeiraea simplicifolia Griffonia simplicifolia* BS I green *Griffonia*-FITC, Sigma-Aldrich, St. Louis, MO, USA in 1:500 dilution for 15 min at 37 °C.

#### Helix pomatia lectin staining

After fixation, permabilization and blocking steps see above MPMVEC cultures were stained with lectin HPA Alexa 488 conjugate Thermo Fischer Scientific in 1:1000 dilution for 1 h.

#### Actin cytoskeleton staining

MPMVEC cultures were challenged with thrombin 50 nM for 30 min or vehicle PBS. After fixation, permabilization and blocking steps see above cells were stained with Texas Red-phalloidin Thermo Fisher Scientific for 1 hour.

For all the experiments coverslips were rinsed with PBS and mounted in ProLong^®^ Gold Antifade Reagent with or without DAPI. All imaging was observed with 200×/400× or with 63× immersion oil objective lenses using a microscope Axiolab; Carl Zeiss, Oberkochen, Germany.

### Gel electrophoresis and western blotting

Cell lysates, 40 μg and 15 μg respectively, were combined with 2× Laemmli sample buffer containing 5% β-mercaptoethanol, and boiled for 8 min at 100 °C. Equal volumes were loaded in each lane of a 4%–20% precast polyacrylamide gel BioRad, Hercules, CA, USA and resolved by sodium dodecyl sulfate-polyacrylamide gel electrophoresis for 1.5 h at 80 V. Then, they were transferred to a 0.2 μm polyvinylidene difluoride membrane Immunblot; BioRad at 40 V for 4 h at 4 °C. The membrane was blocked for 1 h in blocking buffer at room temperature and immunoblotted overnight at 4 °C with primary antibody in blocking buffer. The next day, the polyvinylidene difluoride membrane was washed four times with Tris-buffered saline with Tween5-min each and horseradish peroxidase-conjugated secondary antibody addedfor 1 h. After washing, the immunocomplexes were visualized by enhanced chemiluminescence detection Pierce using a Kodak 440CF Image Station Carestream, Rochester, NY, USA. Equal loading of protein was confirmed by β-actin or glyceraldehyde 3-phosphate dehydrogenase Cell Signaling, Danvers, MA, USA. The intensities of protein signals were quantified based on the optical density of immunoblots using Image J National Institutes of Health, Bethesda, MD, USA.

### Measurement of trans-endothelial electrical resistance TER across EC monolayers

The barrier properties of cell monolayers were characterized using an electric cell-substrate impedance sensing ECIS system Applied BioPhysics, Troy, NY, USA as described previously [12,13]. Cells were grown to confluence in eight-well gold-plated arrays Applied BioPhysics that had been coated with a fibronectin matrix in the same concentration as the cell cultures. The total resistance across the monolayers comprised the resistance generated between the ventral cell surface and the electrode, as well as by the resistance between cells. Initial resistance at the onset of our multiple experiments was normalized to 1. A 4,000-Hz AC signal with 1-V amplitude was applied to the EC monolayer through a 1-MΩ resistor to create an approximately constantcurrent source 1 μA. After a baseline measurement, cells were treated with various concentrations of nocodazole, LPS, adenosine or sphingosine-1-phosphate. Changes in TER in response to the stimuli were recorded in real time. All TER measurements were done in triplicate, and each experiment repeated at least thrice.

## Results

### Production of MPMVECs

**Figure 1:**
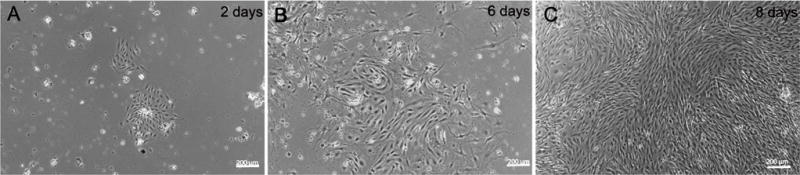
Nesting of mouse pulmonary microvascular ECs. Phase contrast micrographs of mouse pulmonary microvascular endothelial cells culture on (A) day-2 showing Dynabeads® in culture and an inset with a “nest” of endothelial cells and; (B) day-6; (C) Shows a phase-contrast confluent monolayer on day-8. Scale bars are 200 μm.

The harvesting protocol described in the Materials and Methods section enabled consistent isolation of MPMVECs. There is a step by step description of the protocol and the results are documented in our subsequent results and figures. At day two the cells have formed small nests with a typical EC appearance. Dynabeads^®^ are visible in the initial cell clusters Figure 1A. By days 5–6 the cells are developing confluence in the 12-well plates and thereafter they grow rapidly showing 80% to 100% confluence at days 6–8 Figure 1B and Figure 1C. When cells are confluent in 12-well plates at 6–8 days they are ready for passaging. Cells shown are small and varied from a cobblestone appearance to small and slightly elongated monolayers that are homogeneous Figure 1A and Figure 1C.

### Identification of MPMVECs

**Figure 2:**
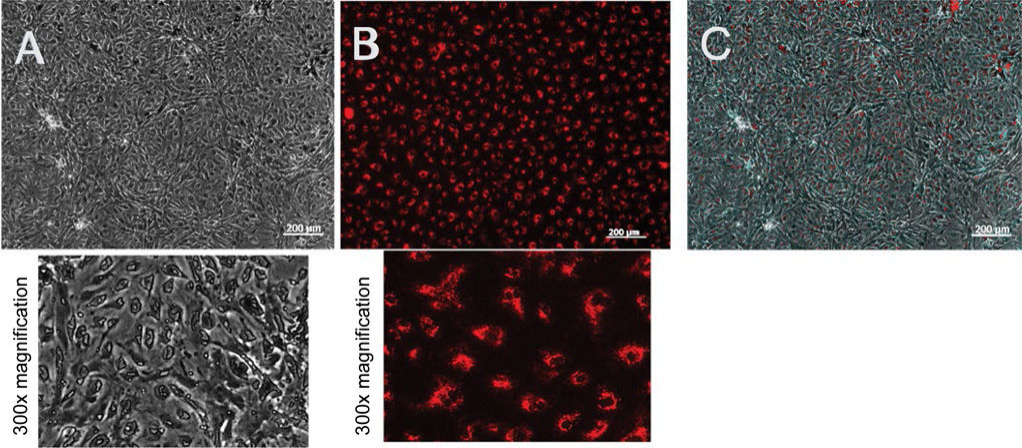
Labeling of mouse pulmonary microvascular endothelial cells with Ac-LDL. Labeling of mouse pulmonary microvascular endothelial cellson day-6 (passage 2) with acetylated-low density lipoprotein cultured at 37 °C in complete endothelial growth basal medium-2 (Lonza, Allendale, New Jersey, USA) on fibronectin (A) Phase-contrast micrograph of a confluent monolayer and below is 300× magnifications; (B) Uptake of acetylated-low density lipoprotein and below is 300× magnification; (C) Confluent monolayer merged with acetylated-low density lipoprotein. Scale bars are 200 μm.

**Figure 3:**
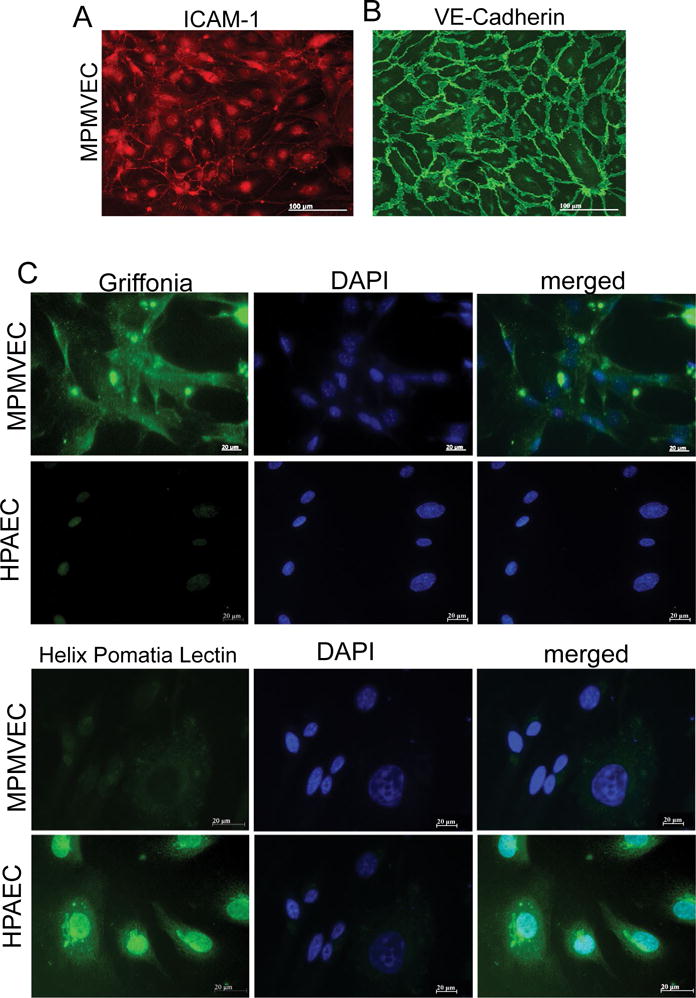
A Characteristics of mouse pulmonary microvascular endothelial cells (MPMVECs). MPMVECs were cultured at 37 °C in complete endothelial growth basal medium-2 on fibronectin until confluent (A) Expression of Intercellular adhesion molecule-1 (ICAM-1) in representative micrograph of cultured endothelial cells; (B) Expression of VE-cadherin is localized at the cell-cell junctions shown in representative micrograph with expression of vascular endothelial cadherin; (C) Endothelial cells isolated from murine lungs or HPAEC stained either with *Griffonia* or *Helix Pomatia* lectin. Scale bars are 20 μm.

Acetylated-low density lipoprotein Ac-LDL was used to identify endothelial cells in our cultures. The cells are shown labeled on day-6 passage 2 with Ac-LDL Figure 2A, Figure 2B and Figure 2C. Ac-LDL is known to be taken up by both macrophages and endothelial cells and can be used to identify these cells from mixed cell populations [14]. Macrophage morphology is easily recognized from ECs and moreover macrophage culture protocols are distinctive from EC culture protocols such as using bone marrow or peritoneal cavity for cultures and alternate culture medium making it separate from the culture of lung microvascular endothelial cells. In addition to Ac-LDL stain the cells expressed the characteristic proteins of VECs: ICAM-1, a cell surface glycoprotein typically expressed on endothelial cells and VE-cadherin an endothelial specific adhesion molecule located at junctions between EC [15] Figure 3A and Figure 3B. *Griffonia simplicifolia* GS1 is a lectin that binds preferentially to microvascular endothelial cells MVEC in mouse tissues and the binding of GS1 provides further evidence that MVEC are present in a highly specific surface glycosylation pattern [16]. To further examine the purity of our murine microvascular endothelial cells, the cells were stained with *Griffonia*-FITC and DAPI Figure 3C. Pulmonary endothelium displays considerable heterogeneity along the vascular axis from arteries to capillaries. GS1 interacts with pulmonary microvascular endothelium and not with extra-alveolar endothelium in both arteries and veins [11,17]. We compared human pulmonary artery endothelial cells HPAEC that do not interact with GS1 to MPMVEC to further evaluate the purity of the cell culture. The results show that GS1 consistently stained the MPMVEC and not the HPAEC providing a specific histochemical marker for our endothelial cell cultures compared to macrovascular cultures. To provide a negative control we labeled MPMVEC and HPAEC with the lectin *Helix pomatia* HPA that preferentially recognizes macrovascular cells [18]. The results show that HPA consistently recognized the HPAEC and not the MPMVEC Figure 3C. The combination of staining with As-LDL, Icam-1, VE-cadherin and the lectin GS1 and no stain or non-specific stain with lectin HPA identifies our cells as microvascular ECs.

**Figure 4:**
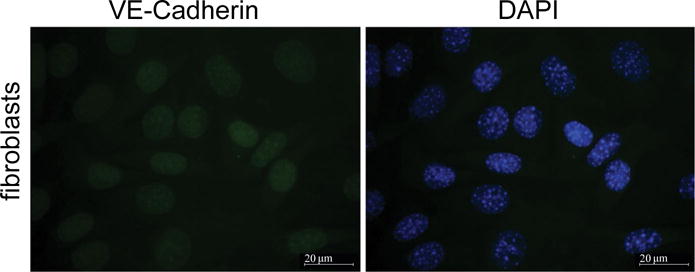
NIH3T3 fibroblasts are stained with DAPI and VE-cadherin. Fibroblasts were stained with cultured NIH3T3 cells grown on glass coverslips. Cells were fixed in a 3.7% formaldehyde solution in PBS for 10 min at and washed three times with PBS. The cells were permeabilized with 0.2% Triton X-100 in TBS supplemented with 0.1% Tween 20 (TBST) for 5 min, washed three times with PBS and blocked with 2% BSA in TBST for 1 h. Incubation with specific antibodies diluted with a blocking solution was performed for 1 h at room temperature. Specific antibody was used to detect VE-cadherin. After three washes with PBS, the cells were incubated with appropriate secondary antibody (1:300) conjugated with fluorescent dye Alexa 488 (green) 1 h at room temperature. The coverslips were mounted with proLong antifade reagent with DAPI. After immunostaining, the cells were analyzed using Zeiss Axiolab microscope using 63 × oil immersion objective. Scale bars are 20 μm.

Common contaminants of endothelial cell cultures are fibroblast cells. To our knowledge no specific markers for fibroblasts exist therefore we stained NIHT3T fibroblasts with VE-cadherin marker and DAPI as a nuclear DNA stainto compare with our cultured MPMVECs with the fibroblast culture. The results of staining the fibroblasts cells with VE-cadherin show that fibroblasts lack the classical demonstration of the intercellular junctions Figure 4A and Figure 4B that we have demonstrated in our murine endothelial cultures. VE-cadherin stain on fibroblasts are compared with Figure 3B that demonstrates clear VE-cadherin outlines that are without gaps or other cells in the MPMVEC.

### Remodeling of actin cytoskeleton by thrombin in MPMVECs

**Figure 5:**
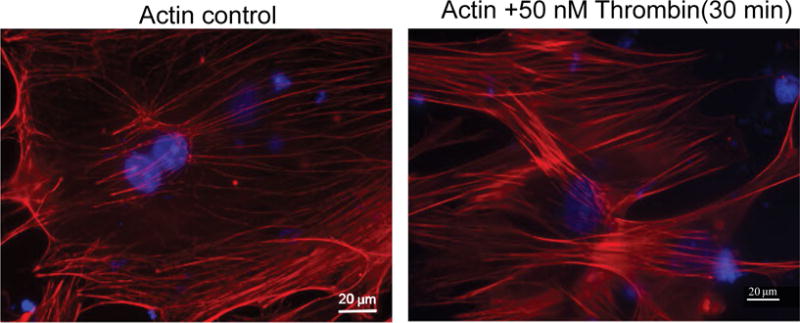
Thrombin treatment induced increased actin cytoskeleton rearrangement in MPMVEC. MPMVECs were cultured at 37 °C in complete endothelial growth basal medium-2 until confluent. The cells grown on coverslips and fixed with 3.7% formaldehyde solution in PBS for 10 min and washed 3 times with PBS and stained with Texas Red®-phalloidin for actin. Untreated cells are compared to thrombin treatment (50 nM) × 30 min. Visualization was performed using microscope (Axiolab; Carl Zeiss, Oberkochen, Germany) at 63 × oil immersion objective. Scale bars are 20 μm.

Thrombin is a known barrier-disruptive agent and pathologically increased thrombin results in EC cytoskeleton remodeling and actin stress fiber formation as well as loss of cell-cell contact in the microvascular endothelium [12]. To examine changes in the MPMVEC actin cytoskeleton in the presence of thrombin we challenged the cells with thrombin 50 nM and observed the EC cytoskeleton. The results demonstrated increased actin stress fiber formation compared to untreated cells Figure 5.

### Barrier properties of MPMVECs

**Figure 6:**
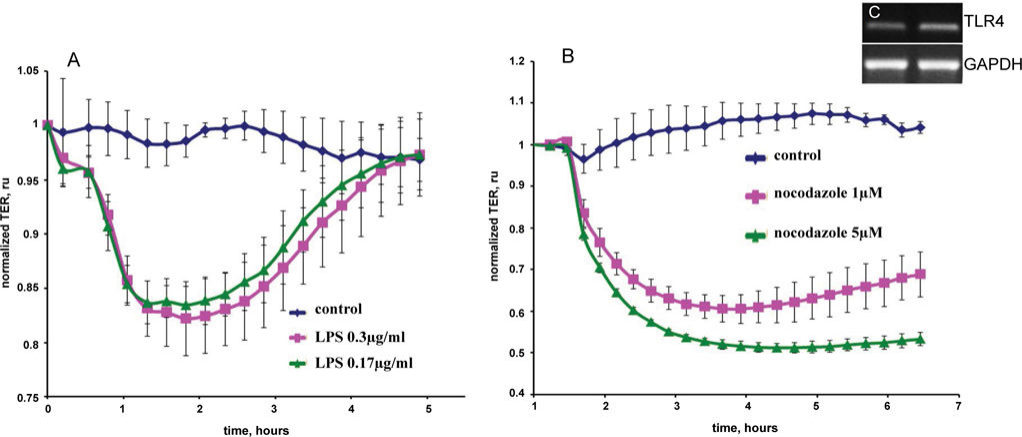
Time-dependent decrease in trans-endothelial resistance (TER) of mouse pulmonary microvascular endothelial cells (A) LPS (0.3 μg/mL or 0.17 μg/mL) and (B) nocodazole (1 μM or 5 μM). Eight-well arrays were inoculated with mouse pulmonary microvascular endothelial cells (50,000); 48–72 h later, confluent monolayers were observed exhibiting TER of ≈800 MΩ to ≈900 MΩ. (A) LPS or; (B) Nocodazole or veh were added and TER values recorded continuously at 20-s intervals over the next 6 h. Data are representative of three separate experiments; (C) PCR analysis for TLR4 expression (Inset) RT-Polymerase chain reaction analysis for toll like receptor-4 (TLR4) expression in endothelial cell from murine lungs. GAPDH expression was used as a housekeeping control.

**Figure 7:**
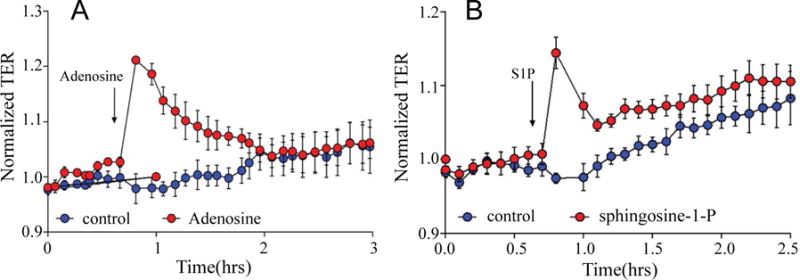
Trans-endothelial resistance (TER) of the barrier protective agents adenosine and Sphingosine-1-phosphate in mouse pulmonary microvascular endothelial cells. Eight-well arrays were inoculated with mouse pulmonary microvascular endothelial cells (50,000); 48–72 h later, confluent monolayers were observed exhibiting TER of ≈800 MΩ to ≈900 MΩ. (A) adenosine orveh; (B) Sphingosine-1-phosphate orveh were added and TER values recorded continuously at 20-s intervals over the next 3 h. Data are representative of three separate experiments.

Forty-eight to seventy-two hours after being seeded on eight-well arrays in preparation for measurements of TER, MPMVECs exhibited a tight monolayer ≈800 MΩ to ≈1000 MΩ. To demonstrate the barrier properties of MPMVECs we tested two substances known to increase the permeability of pulmonary and two EC barrier protective substances. ECs: LPS a Gram-negative product Figure 6A and nocodazole a microtubule-depolymerizing agent Figure 6B. LPS produced a time – and concentration-dependent decrease in TERof MPMVECs. Interestingly, the result of LPS-induced TER was not as robust as that produced in human pulmonary microvascular endothelial cells and the cells returned to baseline after six hours. Nevertheless it is well known that LPS is used in mouse models of sepsis and ARDS. Nocodazole produced a strong decrease in TER that was in accordance with studies using human pulmonary ECs [19] Figure 6B. We performed quantitative reverse-transcription-polymerase chain reaction qPCR analysis for Toll-like receptor TLR-4, the sensor of Gram-negative bacteria endotoxin, to evaluate expression in MPMVEC Figure 6C. Totest the barrier protective properties of MPMVEC we tested adenosine, a well-known lung EC barrier protective agent [7] and spingosine-1-phosphatase S1P, an important lipid mediator that has been shown to augment the pulmonary EC barrier function [20]. The results demonstrate that adenosine and S1P maintained their known protective properties in TER over time Figure 7A and Figure 7B.

## Discussion

Study of the pulmonary endothelial barrier is very important in diseases such as acute respiratory distress syndrome, pulmonary edema, severe pneumonia and other diseases in which the pathogenesis includes loss of integrity of the pulmonary endothelial capillary barrier with increased permeability due to disruption of the microvascular endothelium. Animal models for study of the pulmonary endothelial barrier are well established. Nevertheless, there has been a paucity of studies focusing on MPMVECs *in vitro*.

The difficulty in isolating and growing cells is well known and, although protocols have been published, many are complicated and do not produce consistent results. Here we demonstrated a consistently successful protocol for the culture of MPMVECs. Cells were selected by PECAM, which is highly expressed on ECs [21]. A large portion of the intercellular junction of ECs comprises PECAM and is considered a standard for separation of pulmonary EC [19]. The cells were separated with PECAM conjugated magnetic beads providing an EC selective process. To identify the cells as endothelial cells they were cultured and labeled with acetylated-low density lipoprotein Figure 2 and characterized by immunocytochemistry with ICAM-1 and VE-cadherin Figure 3A and Figure 3B. ICAM-1 is an endothelial and leukocyte associated transmembrane protein that is important in stabilization of EC cell-to-cell interactions and it facilitates leukocyte EC transmigration. ICAM-1 is present continuously in low concentrations in the membranes of EC.ICAM-1 has been shown to be up-regulated in activated EC and has an important role in endothelial transmigration [22]. VE-cadherin is a cell-cell adhesion glycoprotein that is of vital importance for the maintenance and control of EC contacts [15]. VE-cadherin is an early, constitutive and specific marker of endothelial cells [23] and the VE-cadherin antibody detects EC at the cell-to-cell junctions, maintains the cellular junctions and cell adhesion. Our cells are confidently identified as EC by the combination of selective separation, and then identification by Ac-LDL, ICAM-1 and VE-cadherin.

Lectin binding has been utilized as an effective method of discriminating between macro and microvascular endothelial cells [24]. *Griffonia simplicifolia* GS1 lectins bind specifically to endothelial cells and some epithelial cells however they are preferentially expressed in endothelial cells in mouse tissues [16]. GS1 preferentially binds to microvascular endothelium and has been used to identify endothelium in murine models and in human lung MVEC [25,24]. We further identified our cell cultures as microvascular by the expression of GS1 and compared them to a culture of human microvascular pulmonary artery endothelial cells HPAEC which did not express GS1 Figure 3C. The lectin *Helix pomatia* HPA on the other hand preferentially binds to macrovascular endothelium [24]. Therefore we confirmed our cell cultures as microvascular EC by comparing HPA expression in MPMVEC and HPAEC Figure 3C.

Fibroblasts are common contaminants of microvascular endothelial cell cultures [26]. To inspect the purity of our cell cultures we compared them with cultured NIHT3T fibroblasts stained with DAPI and VE cadherin. Figure 4 shows that the NIHT3T cells do not express VE cadherin and only apparent is a small amount of nuclear non-specific stain. This can be compared to the MPMVEC with clear expression of VE-cadherin at the EC junctions Figure 3B. The successful culture of MVEC is very dependent on the sample preparation. We discussed in detail in the methods section the rigorous washing steps that are imperative to avoid cell culture contamination. We found that the washing of the cells during PECAM isolation is the most important step to avoid large cell clumps that could consist of contaminating fibroblasts.

We evaluated the barrier properties of MPMVECs by trans-endothelial resistance TER in Electric cell-substrate impedance sensing ECIS, a non-invasive biophysical approach to monitoring living cells Figure 6A and Figure 6B. We made cDNA and RNA and undertook RT q-PCR for the particular receptor of interest, TLR4 Figure 6C. LPS upon stimulation of TLR4 directly affects endothelial barrier function and promotes hyperpermeability by means of potently activating leukocyte infiltration, inducing oxidative and nitrosative stress [5]. LPS elicited an increase in MPMVEC permeability consistent with known barrier disruption properties [27] Figure 6A. Nocodazole, an inhibitor of microtubule polymerization significantly decreased TER in the MPMVEC, indicating EC barrier dysfunction Figure 6B. It has also been demonstrated that microtubule disruption is linked to decreases in cortical actin and increases in stress fiber formation and endothelial contraction [28]. We furthermore examined the barrier disruptive action of thrombin result with EC immunocytochemistry demonstrated cytoskeleton remodeling and actin stress fiber formation that is consistent with known properties of thrombin [29] Figure 5. MPMVEC barrier protective properties were studied with Adenosine and sphingosine-1-phosphate S1P promoters of endothelial barrier function Figure 7A and Figure 7B. Adenosine exerts barrier-protective effects with respect to lung permeability and injury [7] and SP1 is a key regulator of endothelial barrier function and pharmacological administration of S1P has restored EC barrier function, decreased lung edema and improved survival in murine models [8]. The results show maintenance of the TER in MPMVEC when treated with adenosine and S1P. The present studies suggest that the baseline barrier function of MPMVECs respond to the edemagenic agent LPS and the microtubule-destabilizing agent nocodazole and barrier promoter agents adenosine and S1P *via* ECIS.

An advantage of murine studies is the ability to study *in vitro* cell culture so that the cells alone can be studied apart from the extremely complex multifunctional systems and diverse cells that comprise the intact animal. Hence, a complex barrier is removed, thereby providing a simpler level in which to study the system of interest [30]. Traditionally, creating murine cell cultures is difficult but this method has become routine in our research team.

When we first started the experiments, the initial drawbacks were limited growth of cells, early senescence, and low purity of cell type. As we revised the protocol, these problems disappeared. We froze the cells in liquid nitrogen and thawed them as needed. We were able to undertake ≤ 10 passages but observed senescence after 7–8 passages. We used the cells for experiments within 3 weeks or froze them for future experiments.

We experimented with the matrix using gelatin, collagen and fibronectin. The best results were achieved with fibronectin. Gelatin is less expensive than fibronectin and supports cells. However, passaging was more difficult and there were more problems with senescence. Fibronectin has the advantage in that contaminants bind less readily to it [14].

Complete EBM-2 endothelial basal medium and BulletKit was used for plating cells as well as in all passages and media changes. Many protocols use DMEM, which contains inorganic salts, amino acids, vitamins and glucose. Any growth factors in DMEM must be added. We used DMEM while isolating cells but cells were finally plated with EBM-2. EBM-2 contains in addition to the nutrients noted in DMEM endothelial growth factors such as vascular endothelial growth factor. Vascular endothelial growth factor is involved in the survival, growth, migration and vascular permeability of vascular ECs and is an important component of the *milieu* of ECs. We used penicillin and streptomycin to minimize the risk of cell contamination.

Trypsin is routinely used to lift ECs cells from a cell culture, it is a pancreatic enzyme that breaks down proteins to passage cells or to remove Dynabeads^®^ from cells. We found that the efficiency of the plating and yield of cell passaging was diminished with trypsin. Other scholars have documented very low expression of EC markers such as PECAM due to apparent damage by the enzymatic digestion of trypsin [4]. However, we were able to detach and passage MPMVECs with very little damage using a commercially available cell-dissociation solution. This cell-dissociation solution was developed to detach adherent cells gently and effectively. It had protease and collagenolytic activity, and does not contain mammalian- or bacterial-derived products [31]. In our initial cell isolations using trypsin, the passages often had a low yield and, although they recovered, the time-to-proliferation was longer. Since we started using the cell-dissociation solution for the detachment and passage of cells, cells adhered to the matrix and proliferated immediately. We did not detach Dynabeads^®^ from cells rather as the cells proliferate and were passaged the number of Dynabeads^®^ dwindled.

In our initial experiments, we plated cells in large T-75 flasks or 100 mm dishes. However, we had improved cell-cell contact, morphology and proliferation when we employed 12-well dishes. We used 1–2 lungs in each dish. They reached confluence in 6–7 days and, once they were passaged into a larger plate, they propagated rapidly and we had many cells for experimentation.

As the cultures grew ifproliferation of fibroblasts or smooth muscle cells was observed, a second isolation with Dynabeads^®^ was undertaken to improve cell purity. We noted that the initial isolation and wash with the beads on the magnet with moderately vigorous titration with a 1 mL pipette was the most important step to minimize “clumps” of cells that might trap contaminating cells. We improved with experience and we subsequently have minimal contamination in our cultures. Other authors have reported manual methods to remove contaminant colonies using Pasteur pipettes or cell scrapers [30]. We did not use these methods because a second isolation proved sufficient.

We have used mice ranging in age from 7 days to 2 months, and found that the protocol works well at all ages. It appears that the preferred age for our protocol is 3–5 weeks. Younger mice seem to have more progenitor cells and the cells of older mice work efficiently but we have not been able to passage them as long.

We have included a table of the products and suppliers that we use Table 1. This protocol is easy to replicate and is efficient. The results can be used to obtain experimental data in murine models.

## Figures and Tables

**Table 1:** Products and supplies for culture of mouse pulmonary microvascular endothelial cells.

Product	Supplier	Supplier address	Product number
Dulbeccos’s modified Eagles’s medium (DMEM), powder, high glucose	Thermo Fisher Scientific	West Columbia, SC, USA	12100-046
Fetal bovine serum (FBS)	Sigma-Aldrich	St. Louis, MO, USA	SV30014.03 or F0926
Endothelial basal medium-2 (EBM-2) and BulletKit™	Lonza	Allendale, NJ, USA	CC-3156
Penicillin and streptomycin	Thermo Fisher Scientific	As above	15140122
Fibronectin	Thermo Fisher Scientific	As above	33016015
Gelatin	Sigma-Aldrich	As above	G1890
Collagen type I (PureCol)	Advanced BioMatrix	San Diego, CA, USA	5005-100 ML
Hank’s balanced salt solution (HBSS)	Thermo Fisher Scientific	As above	14185052
Phosphate-buffered saline (PBS) without Ca^2+^ and Mg^2+^	Thermo Fisher Scientific	As above	14190250
Bovine serum albumin (BSA)	Thermo Fisher Scientific	As above	A9647
Collagenase type I	Worthington Biochemical	Lakewood, NJ, USA	LS004196
Dynabeads^®^ sheep anti-rat IgG	Thermo Fisher Scientific	As above	11035
DynaMag^®^ spin magnet (MPC)	Thermo Fisher Scientific	As above	12320D
Platelet endothelial cell adhesion molecule (PECAM-1; CD31) *discontinued at TFS May consider other company	Santa Cruz	Santa Cruz, CA, USA	Sc-376764
Cell strainer (70 μm)	Thermo Fisher Scientific	As above	22-363-548
Sterilization filter (0.22 μm)	Thomas Scientific	Swedesboro, NJ, USA	1226S77
0.05% Trypsin-EDTA	Thermo Fisher Scientific	As above	25300-054
Accutase	Innovative Cell Technologies	San Diego, CA, USA	#AT-104
ECIS array	Applied BioPhysics	Troy, NY, USA	8W1E PC

## Abbreviations

ECs: Endothelial cells
ARDS: Acute respiratory distress syndrome
MPMVECs: Mouse pulmonary microvascular endothelial cells
qPCR: Quantitative reverse-transcription-polymerase chain reaction
ECIS: electric cell-substrate impedance sensing
TLR4: Toll like receptor 4
VE-cadherin: Vascular endothelial cadherin
Ac-LDL: Acetylated-low density lipoprotein
ICAMs: Intercellular adhesion molecules
GS1: *Griffonia simplicifolia*
HPA: Helix pomatia
TER: Transendothelial electrical resistance
LPS: Lipopolysaccharide
PECAM: Platelet endothelial cell adhesion molecule
^®^: Registered trademark
MPC: Magnetic particle concentrator
DBS: Dynabead^®^ solution
HBSS: Hank’s balanced salt solution
HPAE: Chuman pulmonary artery endothelial cells
S1P: Sphingosine-1-phosphate
EBM-2: Endothelial basal medium
DMEM: Dulbecco’s modified eagle’s medium
